# Comparison of antibody responses against *Mycobacterium tuberculosis* antigen Rv0679c in tuberculosis patients from the endemic and non-endemic regions of the Beijing genotype: a case control study

**DOI:** 10.1186/s12879-017-2442-5

**Published:** 2017-05-15

**Authors:** Jingge Zhao, Takashi Matsuba, Xiaoyan Zhang, Susan Leano, Chie Nakajima, Haorile Chagan-Yasutan, Elizabeth Freda Telan, Yasuhiko Suzuki, Toshio Hattori

**Affiliations:** 1 Laboratory of Disaster Medicine, International Research Institute of Disaster Science, Tohoku University, Sendai, Miyagi 980-8574 Japan; 2 Division of Bacteriology, Department of Microbiology and Immunology, Faculty of Medicine, Tottori University, Yonago, Tottori, 683-8503 Japan; 3 Shanghai Public Health Clinical Center, Fudan Univeristy, Shanghai, 201508 China; 4 STD AIDS Cooperative Central Laboratory, San Lazaro Hospital, Quiricada Street, 1003 Manila, Philippines; 5 Division of Global Epidemiology, Hokkaido University Research Center for Zoonosis Control, Sapporo, Hokkaido 001-0020 Japan; 6 The Global Station for Zoonosis Control, Hokkaido University Global Institution for Collaborative Research and Education, Sapporo, Hokkaido 001-0020 Japan; 7 Graduate School of Health Science Studies, Kibi International University, 8 Igamachi, Takahashi, 716-8508 Japan

**Keywords:** *Mycobacterium tuberculosis*, Dimorphic antigen, Rv0679c, Beijing genotype, Philippines, Immunoglobulin G, Immunoglobulin A

## Abstract

**Background:**

Strains of the Beijing genotype of *Mycobacterium tuberculosis* (MTB) are reportedly associated with the virulence of tuberculosis (TB) infection, unfavorable outcomes of anti-TB treatment, and the global TB pandemic. Rv0679c, a hypothetical membrane protein related to host cell invasion, has a Beijing genotype-specific mutation at residue 142 (Asn142Lys). Antigenicity differences between Rv0679c-Asn142 (N-type) and Rv0679c-Lys142 (K-type) have been previously observed in mice antigen-antibody responses. However, the immune response to Rv0679c in humans remains unknown. Therefore, we aimed to investigate the anti-Rv0679c immune response in TB patients from the endemic and non-endemic regions of the Beijing MTB genotype.

**Methods:**

We analyzed the Rv0679c-specific antibody responses in 84 subjects from the endemic region of the Beijing genotype MTB in China, including 45 pulmonary TB patients (C-PTB) and 39 healthy controls (C-HC), and 81 subjects from the Philippines (the endemic region of the non-Beijing genotype), including 51 pulmonary TB patients (P-PTB) and 30 healthy controls (P-HC). Anti-tuberculous-glycolipid (TBGL) antigen was used as the control antibody.

**Results:**

TBGL IgG titers were higher in both C-PTB and P-PTB than those in their corresponding HC (C-PTB median 4.2, P-PTB median 11.2; C-PTB vs. P-PTB, *p* > 0.05), suggesting immune response comparability in PTB from two different countries. C-PTB showed a higher response compared to C-HC for anti-K-type IgG (53.3%) than anti-N-type IgG (6.67%); this response was not observed in P-PTB (both N-type and K-type 9.80%).

**Conclusion:**

Dimorphic antigen Rv0679c was found to be associated with distinct immune response patterns, indicating the role of Beijing/non-Beijing genotype of MTB in stimulating specific responses in TB patients from the endemic region of Beijing MTB. Meanwhile, reactions to Rv0679c in patients and HC from non-endemic regions of the Beijing MTB may be caused by the response to the common epitope of Rv0679c N/K-type.

## Background

Tuberculosis (TB) remains a global health problem. In 2015, there were an estimated 10.4 million new cases and 1.4 million deaths due to TB, worldwide [1]. Although China ranks third, after India and Indonesia, in terms of TB incidence, the estimated mortality rate of TB in China is 2.7 per 100, 000 cases, which is lower than that in the Philippines (14.3 deaths per 100,000 cases) [1]. By 2012, the prevalence rate of TB was 291/100,000 in the eastern, 463/100,000 in the middle, and 695/100,000 in the western parts of China [2]. Meanwhile, by 2007, in the Philippines, the prevalence rate of TB was 200/100,000 for smear-positive TB and 470/100,000 for culture-positive TB, with an increasing trend in the incidence of TB infection [3]. These surveys collectively indicated the significance of TB in both countries. However, the Beijing genotype of *Mycobacterium tuberculosis* (MTB), a clade that originated in East Asia and led to the occurrence of epidemics [4], dominates TB infection in China in contrast to the non-Beijing genotypes of MTB prevalent in the Philippines [5]. The Beijing genotype of MTB was first identified by van Soolingen et al. [6], and the worldwide spread of strains with this genotype could be attributed to the lower efficacy of Bacillus Calmette–Guérin (BCG) vaccination, multi-drug resistance, and high virulence [7–10]. According to a national survey in China using 4017 samples in 2012, 53.2% and 76.5% of MTB infections in southern and northern China, respectively, were due to strains of the Beijing genotype [11]. A recent survey conducted in northeastern China indicated a predominance of the Beijing genotype, accounting for 89.5% of TB infections [12].

In MTB strain H37Rv, the Rv0679c protein, with a predicted molecular mass of 16,586 Da (Da), consists of 165 amino acids and includes an N-terminal signal sequence and a consensus lipoprotein-processing motif. The entire nucleotide sequence of *Rv0679c* is well conserved in the MTB complex (MTC). Rv0679c is reportedly involved in the process of MTB entrance into host cells [13]; however, its functions and contributions to MTB-host interactions remain unclear. Recently, a single nucleotide polymorphism, C426G, has only been found in *Rv0679c* in isolates identified as members of the Beijing genotype family. This polymorphism involves a single amino acid substitution of asparagine (Asn) with lysine (Lys) at residue 142 [4]. Therefore, Rv0679c-Asn142 (N-type) is unequivocally associated with non-Beijing genotypes of MTB and *Mycobacterium bovis* (including the BCG strain), while Rv0679c-Lys142 (K-type) is found exclusively in the Beijing genotype of MTB, including ancient and modern sublineages [4]. Mouse monoclonal antibodies (mAbs) specifically against Asn142 or both Asn142 and Lys142 have been produced, whereas no such monoclonal antibody reacting specifically against the K-type has been produced [8]. Therefore, consequential effects of Asn142Lys substitution have not been determined. Asn to Lys substitution has been achieved artificially in *Mycobacterium smegmatis* and a significant decrease in detectability of the immune response has been observed [14]. Moreover, a recent study on 20 patients infected with strains belonging to the Beijing genotype and 16 patients infected with those of the non-Beijing genotype of TB from Japan revealed that more patients belonging to the former and latter groups carried high titers of IgG against the K-type and N-type, respectively [15]. To investigate the anti-Rv0679c immune response in TB patients from the endemic and non-endemic regions of the Beijing MTB genotype, we measured specific IgA and IgG antibody titers using enzyme-linked immunosorbent assay (ELISA), in which mouse mAb served as the positive control for N-type and K-type responses. Using a case-control study, we observed a distinct anti-Rv0679c IgG pattern in the samples from Beijing genotype MTB endemic region, and we described different IgG and IgA responses against dimorphic Rv0679c for the first time.

## Methods

### Purification of recombinant Rv0679c proteins and mAbs

Sequences encoding N-terminal truncated forms of Asn142 and Lys142 were amplified by polymerase chain reaction from genomic DNA isolated from non-Beijing and Beijing genotype MTB clinical isolates, respectively. Hybridoma culture supernatants containing anti-Rv0679c mAbs 5D4-C2 and 8G10-H2 were used in this study. Recombinant protein purification and mAb establishment were performed as described previously [8]. Peptide ELISA was performed using Peptide Coating Kit (Takara Bio, Inc., Otsu, Japan). The plate was coated with 16 synthesized peptides, each of which contained 20 amino acids and 10 overlapping amino acids for epitope mapping of the 165 amino acid sequence of Rv0679c (Table 1). The standard provided in the tuberculous glycolipid (TBGL) kit (Kyowa Medex Co., Ltd., Tokyo, Japan) was used to quantify the monoclonal antibody reaction. The experiment was repeated and mAbs supernatant was diluted by a factor of 50 to give an optical density (OD) value of 2, which corresponded to 32 U/mL of the TBGL standard processed under identical experimental conditions. mAbs 5D4–2 and 8G10–2 were used in experiments at this concentration (1/50 dilution). Horseradish peroxidase (HRP)-conjugated goat anti-mouse IgG (Santa Cruz Biotechnology, Inc., Santa Cruz, CA, USA), diluted 1:10,000, was used as the secondary antibody. The reaction patterns of mAbs and recombinant proteins were investigated by western blot. Purified recombinant Asn142 or Lys142 proteins (5 μL of 100 μg/mL) were mixed with equal volumes of sodium dodecyl sulfate-polyacrylamide gel electrophoresis (SDS-PAGE) loading buffer or blue native-PAGE (BN-PAGE) Buffer (Invitrogen, Inc., Carlsbad, CA, USA). Protein and SDS-PAGE loading buffer mixtures were heated at 95 °C for 10 min and electrophoresed using 4–15% SDS-polyacrylamide gel, while proteins and BN-PAGE buffer mixtures were electrophoresed using 4–16% BN-polyacrylamide gel (Invitrogen, Inc.). Separated proteins were stained with Coomassie Brilliant Blue and transferred to polyvinylidene fluoride membranes for western blotting. Membranes were blocked by the Blocking One reagent (Nacalai Tesque, Inc., Kyoto, Japan) for 1 h at room temperature, followed by incubation with a 50-fold dilution of hybridoma culture supernatant containing mAbs in 1% (*w*/*v*) bovine serum albumin (BSA) pH 7.4 or a 20-fold dilution patient’s plasma in fetal bovine serum (FBS) (Wako Pure Chemical, Ltd., Japan) pH 7.4 overnight at 4 °C. After 4 washes with 0.5% Tween-20 in phosphate-buffered saline (PBS), pH 7.4, membranes were incubated with 1:5000 diluted HRP-conjugated goat anti-mouse IgG (Santa Cruz Biotechnology, Inc.) in 1% BSA (*w*/*v*) in PBS pH 7.4 for mAb or HRP-conjugated IgG heavy chain goat anti-human polyclonal antibody (LSBio, Inc. Seattle, WA) in 20% horse serum as the secondary antibody for patient. After 1 h incubation of secondary antibody at room temperature on a shaker, 4 washes were applied prior to visualizing the reactive bands by using Bio-Rad’s versatile ChemiDoc MP system (Bio-Rad Laboratories, Hercules, CA, USA).

**Table 1:** Peptide Numbering and Sequence

Peptide	Number	Sequence
Rv0679c (1–20)	1	H-MVEKPLRADRATHSRLATFAEEEKKK-OH
Rv0679c (11–30)	2	H-ATHSRLATFALALAAAALPLEEEKKK-OH
Rv0679c (21–40)	3	H-LALAAAALPLAGCSSTANPPEEEKKK-OH
Rv0679c (31–50)	4	H-AGCSSTANPPAATTTPATATEEEKKK-OH
Rv0679c (41–60)	5	H-AATTTPATATTTTATSGPTAEEEKKK-OH
Rv0679c (51–70)	6	H-TTTATSGPTAAPTVTTGESTEEEKKK-OH
Rv0679c (61–80)	7	H-APTVTTGESTTASIQIGDMLEEEKKK-OH
Rv0679c (71–90)	8	H-TASIQIGDMLTYGSIGTTATEEEKKK-OH
Rv0679c (81–100)	9	H-TYGSIGTTATLDCADGKSLNEEEKKK-OH
Rv0679c (91–110)	10	H-LDCADGKSLNVAGSDNTLTVEEEKKK-OH
Rv0679c (101–120)	11	H-VAGSDNTLTVNGTCETVTVG-OH
Rv0679c (111–130)	12	H-NGTCETVTVGGANNKIAFDR-OH
Rv0679c (121–140)	13	H-GANNKIAFDRIDERLWVGLEEEKKK-OH
Rv0679c (131–150)	14	H-IDERLVVVGLDNTVTYKNGDEEEKKK-OH
Rv0679c (141–160)	15	H-DNTVTYKNGDPTIDNLGAGNEEEKKK-OH
Rv0679c (146–165)	16	H-YKNGDPTIDNLGAGNRINKEEEEKKK-0H
Rv0679c 142 N-- > K (131–150)	17	H-IDERLWVGLDKTVTYKNGDEEEKKK-OH
Rv0679c 142 N-- > K (141–160)	18	H-DKTVTYKNGDPTIDNLGAGNEEEKKK-OH

### Subjects

Blood samples were collected from adult participants (age > 18 yrs.) recruited from Shanghai Public Health Clinical Center (SHAPHC), affiliated with Fudan University (Shanghai, China), and San Lazaro Hospital (Manila, Philippines). Informed consent was obtained from all participants prior to blood sample collection. The study protocol was approved by the ethics committees of SHAPHC (20,120,706; China), San Lazaro Hospital (20,110,706; Philippines), and Tohoku University School of Medicine (20,121,322; Japan). All active TB (ATB) subjects were diagnosed following the WHO guidelines on screening active TB [16]. Blood samples were collected and stored before initiating anti-TB therapy or under treatment less than 2 weeks. All ATB subjects were tested for antibodies to HIV-1 to exclude immunocompromised individuals from the study. The sputum samples from Filipino TB patients was obtained and tested for genotype of MTB as described in previous [17]. The spoligointernational type (SIT) was determined by comparing the spoligotypes against the international spoligotyping database (SpolDB4) [18]. Healthy control participants (HC) were recruited from laboratory or health care workers at the annual checkup from SHAPH (China) and San Lazaro hospital (the Philippines). All HC were confirmed by chest X-ray as free from ATB, and were tested as HIV negative and free from other infectious diseases at routine blood test, following the policy of each hospital to eliminate the nosocomial infection. The final analysis of HC samples only involved TB antibodies that we had proposed to the ethic committees in each country. A previous studied Beijing genotyp infectous (SIT 1, SpolDB4) subject from Japan was also included as a validation [15]. All of the procedures were conducted in accordance with the Declaration of Helsinki. (Table 2).

**Table 2:** Demographic Information

Parameter		Chinese Subjects		Filipino Subjects	
		C-PTB	C-HC	P-PTB	P-HC
No. of Subjects		45	39	51	30
Age (in years; mean ± SD)^a, b^		60.47 ± 18.19	29.9 ± 6.38	41.3 ± 13.0	33.7 ± 11.0
Sex (no. of males/no. of females)^c^		35/10	28/11	36/15	9/21
TB Antibody Test (positive results)	anti-TBGL IgG^d^	31 (68.9)	7 (17.9)	41 (80.4)	16 (53.3)
	anti-TBGL IgA^e^	21 (46.7)	4 (10.3)	34 (66.7)	10 (33.3)

C-PTB, C-HC, pulmonary TB patients and healthy controls from China; P-PTB, P-HC, pulmonary TB patients and healthy controls from the Philippines

^a^older age in C-PTB than in the others

^b^younger age in C-HC than in C-PTB or in P-PTB

^c^larger proportion of female subjects recruited in P-HC compared to the others

^d^lower positivity of anti-TBGL IgG in the C-HC compared to the others

^e^lower positivity of anti-TBGL IgA in C-HC compared to that in C-PTB or in P-PTB

### Enzyme linked immunosorbent assay

**Fig. 1:**
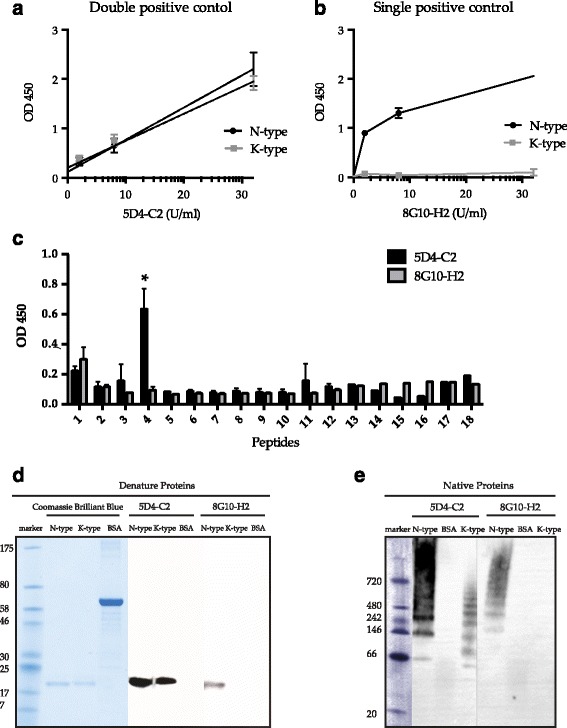
Anti-N/K type or peptides monoclonal antibody responses examined by enzyme-linked immunosorbent assay (ELISA) or western blotting**. a** mAb 5D4-C2 reacted to both N-type and K-type Rv0679c equally, as shown by ELISA. **b** mAb 8G10-H2 reacted to N-type exclusively, as shown by ELISA. **c** 5D4-C2 recognized the linear peptide (peptide-4) covering residues 31–50 (AGCSSTANPPAATTTPATAT) within 16 consecutive overlapping peptides from 1 to 165 of Rv0679c; * indicates a significant difference in the level of reaction between 5D4-C2-peptide-4 and others, *p* < 0.05. **d**, **e** indicates sodium dodecyl-polyacrylamide gel electrophoresis (SDS-PAGE) western blots and native PAGE western blots, respectively, 5D4-C2 reacted with both N-type and K-type, while 8G10-H2 reacted only with N-type; different migration patterns of N-type and K-type were assessed in native PAGE; BSA, bovine serum albumin

Nunc MaxiSorp plates (Thermo Fisher Scientific, Inc., Waltham, MA, USA) were coated with 100 μL/well of purified recombinant Rv0679c N-type and K-type proteins in 0.05 M carbonate-bicarbonate, pH 9.6, and incubated overnight at 4 °C. Optimal amounts of Rv0679c per well for IgA and IgG detection were determined to be 0.15 μg (1.5 μg/mL) and 0.02 μg (0.2 μg/mL), respectively. The supernatant containing mAbs, 5D4-C2 and 8G10-H2 diluted 1:50 in 1% BSA (*w*/*v*) 1 × PBS pH 7.4, were used as anti-Rv0679c IgG controls (Fig. 1), while one sample that responded highly to both N-type an K-type was designated as the anti-Rv0679c IgA positive control. Coated plates were blocked with 3% (*w*/*v*) BSA in PBS pH 7.4 for 6 h at 4 °C. Plasma samples were diluted 100 times by using fetal bovine serum (FBS) (Wako Pure Chemical, Ltd., Japan) that was adjusted to pH 7.4 following the formula of tris aminomethane 6.01 g/L, glucose 18.0 g/L, NaCl 86.67 g/L, and hydrochloric acid (for pH adjustment), and then were added to pre-coated well for 1 h incubation at 37 °C. After being washed 4 times with PBS-0.05% Tween 20 (PBST), HRP-conjugated goat anti-human IgG heavy chain polyclonal antibody was added as the secondary antibody (LifeSpan BioSciences, Inc., Seattle, WA, USA) to detect anti-Rv0679c IgG that was diluted 1:10,000 in 1% (*w*/*v*) BSA in 1 × PBS pH 7.4. HRP-conjugated rabbit anti-human IgA was mixed with unconjugated rabbit anti-human IgA (Agilent Technologies, Santa Clara, CA, USA) at a ratio of 7.5:1 and diluted 1:10,000 in 20% (*v*/v) horse serum in 1 × PBS (pH 7.4) to reduce the background interference for anti-Rv0679c IgA ELISA after wash. The condition for secondary antibody incubation is 1 h at 37 °C. Reactions were visualized using a TMB HRP substrate kit (KPL, Inc., Gaithersburg, MD, USA). Thirty minutes incubation at RT was applied for color development, and values were measured at 450 nm. Levels of TBGL IgG and TBGL IgA antibody controls were measured in plasma using a Determiner TBGL Antibody ELISA kit (Kyowa Medex Co., Ltd., Tokyo, Japan; Table 2). This assay uses TBGL antigens purified from MTB H37Rv and has been described previously [19]. All samples were assayed in a randomized double-blind manner.

### Statistical analysis

The cutoff for identification of samples that were positive for anti-Rv0679c antibody was calculated as the mean OD plus 3 standard deviations of the group with the lowest reaction, which was assigned to C-HC [20]. The cutoff OD for anti-N-type IgG and anti-K-type IgG were set at 0.51 and 0.65, respectively. Despite a higher cutoff value for anti-K-type IgG, no significant difference was observed in the IgG response between Rv0679c types in the C-HC group (data not shown, *p* > 0.05). OD cutoff for both anti-N-type or anti-K-type IgA was 0.39. Data were analyzed using GraphPad Prism 6.0 (GraphPad Software, San Diego, CA, USA). The Kruskal-Wallis test was used to evaluate differences when more than 2 groups were involved. Dunn’s post-hoc test was used to evaluate the differences between the 2 groups following the Kruskal–Wallis test. Chi-square test was used to test variances among groups shown in Table 2 and Fig. 3. Correlations between results were assessed using Spearman correlation coefficients. The results were considered significant at *p* < 0.05. The prediction of 3D structure was achieved on SWISS-MODEL (University of Basel, The Center for Molecular Life Sciences.).

## Results

### mAb and Rv0679c

mAb 5D4-C2 recognized both N-type and K-type, while 8G10-H2 recognized only the N-type (Fig. 1a–e). Epitope mapping by peptide ELISA showed that 5D4-C2 reacted with a peptide (Peptide no. 4 on the X-axis in Fig. 1c) belonging to a common region of Rv0679c. Meanwhile, 8G10-H2 failed to recognize a linear structured peptide (Fig. 1c). In native western blots, Asn142 showed a ladder of higher molecular weight-bands compared to those shown by Lys142 (Fig. 1e, 5D4-C2). However, the 5D4-C2 reactivity against N-type and K-type appeared to be similar under denaturing conditions (Fig. 1d).

### Anti-TBGL antibody as control test

**Fig. 2:**
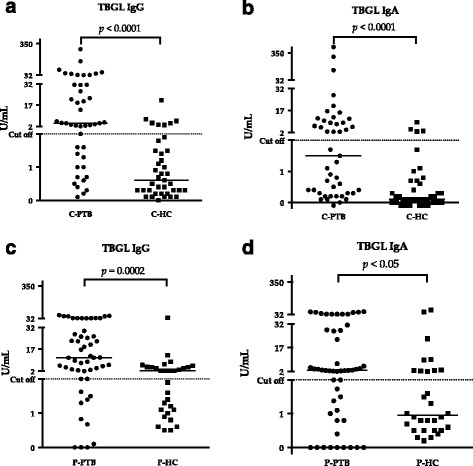
Anti-TBGL antibody response. **a**, **b**, anti-TBGL antibodies between patients and healthy controls from China; **c**, **d**, anti-TBGL antibodies between patients and healthy controls from the Philippines. Cutoff was set to 2 in accordance with the instructions provided by TBGL kits. A significant difference indicated by non-parametric *t*-test was shown as *p* < 0.05

Anti-TBGL IgG and IgA titers were high in patients from China and the Philippines compared to those in their corresponding healthy controls (*p* < 0.05; Fig. 2a–d). In China, 17.9% and 10.3% of HC were positive for anti-TBGL IgG and IgA, respectively, which is significantly lower than the corresponding 53.3% and 33.3% HC from the Philippines (Chi-square test *p* < 0.05, Table 2).

### Anti-Rv0679c antibodies in human serum

**Fig. 3:**
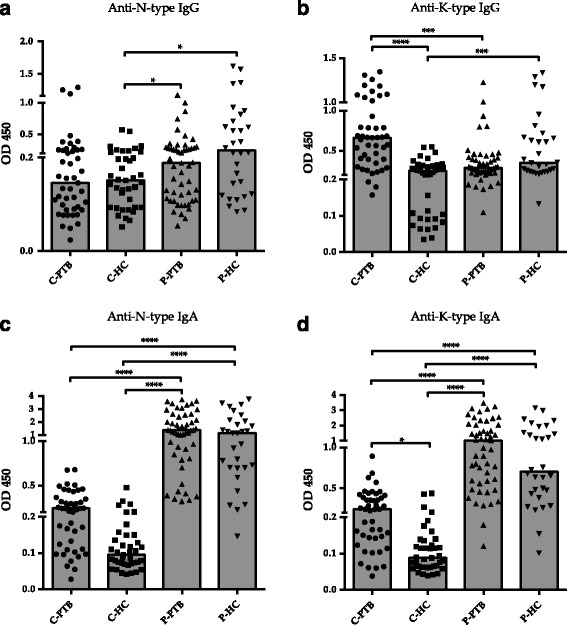
Anti-Rv0679c antibodies in PTB patients from China and the Philippines. C-PTB and C-HC, pulmonary TB patients and healthy controls from China; P-PTB and P-PTB, pulmonary TB patients and healthy controls from the Philippines. Following the Kruskal-Wallis and Dunn’s tests, *, **, ***, **** indicates *p* < 0.05, *p* < 0.01, *p* < 0.005, *p* < 0.0001 between 2 implied groups, respectively. Panels, the medians of indicated groups

Samples from the Philippines displayed higher titers of anti-N-type IgG, compared to the C-HC from China (Fig. 3a). C-PTB showed a higher anti-K-type IgG response compared to C-HC, or P-PTB from the Philippines (Fig. 3b). Filipino participants also showed higher N-type/K-type IgA responses compared to their Chinese counterpart (Fig. 3c and d). Of note, compared to C-HC, C-PTB showed a higher anti-K-type IgA response (Fig. 3d) and an absence of anti-N-type IgA response (Fig. 3c).

### Difference in N-type and K-type specific antibody responses

**Fig. 4:**
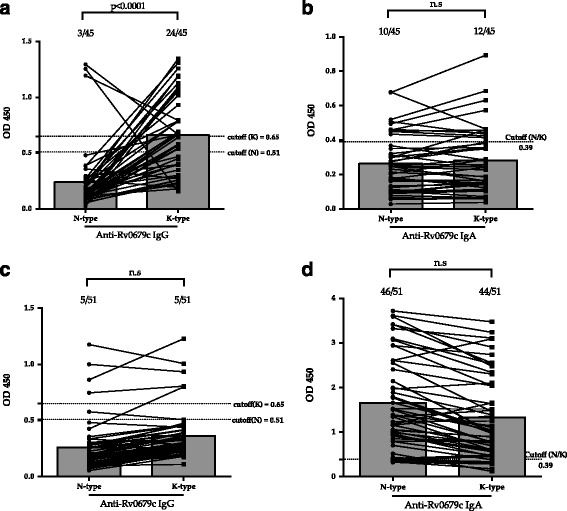
Difference in anti-N-type and anti-K-type Rv0679c specific antibody responses. **a** and **b** refers to the IgG and IgA responses of C-PTB against N- and K-types, respectively; **c**, **d** refers to the IgG and IgA responses of P-PTB against N- and K-types, respectively, respectively. Panels, the medians

The majority of the C-PTB samples were positive for a K-type IgG response than an N-type IgG response (Chi-square, *p* < 0.05). However, no difference in anti-N/K-type IgA responses was observed in spite of 12 samples testing positive for anti-K-type IgA and 10 samples testing positive for anti-N-type IgA (Fig. 4a and b). Interestingly, the response of anti-Rv0679c IgG in C-PTB appeared antithetical (Fig. 4a); namely, those with high anti-K-type IgG levels appeared to be low in anti-N-type IgG, and vice versa. While the antibody responses against N/K-types showed no statistical difference for P-PTB samples, 46 samples were positive for N-type IgA compared to 44, which were positive for K-type IgA (Fig. 4d).

### Correlation of antibody responses in TB patient groups

**Fig. 5:**
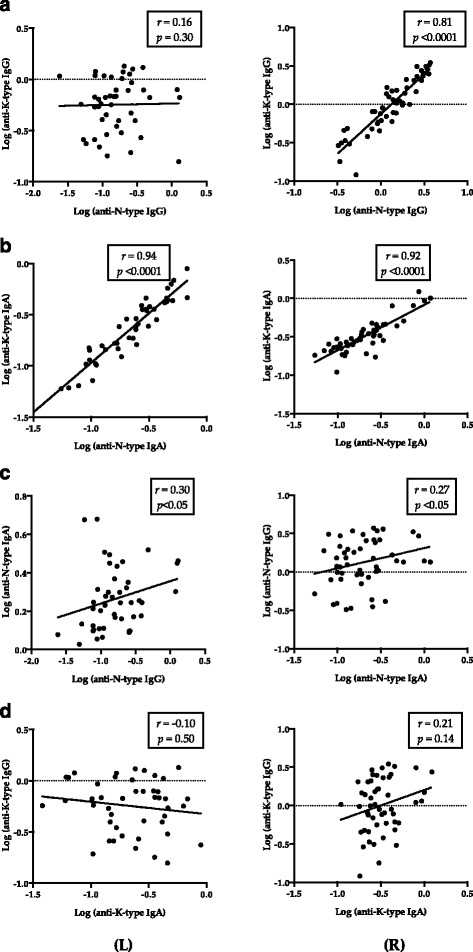
Relationship between anti-Rv0679c specific antibody responses. *L* (Left column): PTB subjects from China; *R* (Right column): PTB Patients from the Philippines. Each dot represents a PTB subject. Correlation graph was prepared based on the log-transferred data of each subject. Spearman analysis was used to calculate *r* and p; *p* < 0.05: significant correlation

No significant correlation was observed between the anti-N-type and anti-K-type IgG responses in C-PTB (Fig. 5a; *r* = 0.16, *p* = 0.30), while a significant correlation between those was found in P-PTB (Fig. 5a; *r* = 0.71, *p* < 0.0001). Significant correlations were observed between anti-N-type IgA and anti-K-type IgA responses and between anti-N-type IgA and anti-N-type IgG responses in C-PTB or P-PTB samples (Fig. 5b–p < 0.05). However, no significant correlation was seen between anti-K-type IgG and anti-K-type IgA in C-PTB or P-PTB samples (*p* > 0.05; Fig. 5d).

### Plasma reaction to K-type under native condition

**Fig. 6:**
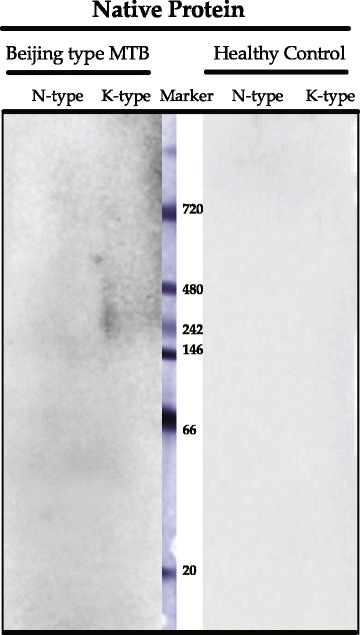
Blue-native PAGE Western blot of human sample. Patient sample T9 reacted with Rv0679c K type only on BN-PAGE, while healthy control sample did not react with either K type or N type

A Beijing type MTB infected Japanese patient exhibited a high reaction against Rv0679c K-type and low reaction against N-type [15]. Western blot for SDS and BN-PAGE were run to investigate antibody affinity under denature and native state of protein, respectively. No blotting bands were detected on SDS Page western blot, but a reaction was detected on BN-Page western blot with a reactive peak at 242–480 kDa (Fig. 6, Beijing type MTB).

## Discussion

A mutated gene leading to the Lys142 variant of the Rv0679c protein had been found in clinical isolates of members of the Beijing genotype family of MTB [4]. In agreement with previous results [14], mAb 5D4-C2 was found to react with both types of purified recombinant Rv0679c by recognizing a linear epitope, identical between N-type and K-type, at the N-terminus of Rv0679c, while mAb 8G10-H2 reacted only with the N-type (Fig. 1a and b). Therefore, 5D4-C2 and 8G10-H2 served as positive controls for anti-K-type IgG and anti-N-type IgG responses, respectively.

**Fig. 7:**
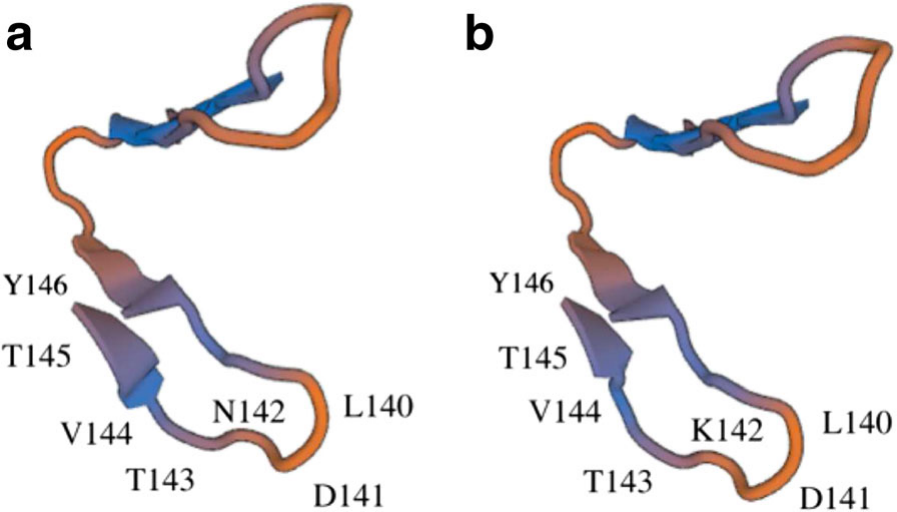
Predictive 3D structure model for Rv0679c N/K type. **a** Rv0679c N type. **b** Rv0679c K type

Reduction of protein aggregation is associated with reduced immunogenicity [21]. Self-aggregation of recombinant Rv0679c-Asn142 protein has been observed in our previous study [8] and was confirmed, in this study, by native polyacrylamide gel electrophoresis conditions and western blotting (Fig. 1e). Lysine and Asparagine are both hydrophilic amino acid while Lys is positively charged, and the difference may explain aggregation of Rv0679c-Asn142 (N-type) [22], which may induce alternative conformational epitope because of structure changes. Swiss-model generated 3D model from amino acids 115 to 146 by detecting gingipain R2 as a template along with 20% sequence identity (Fig. 7a and b). The Asn142Lys substitution could alternate the structure from the turn (L140-T143) to the following beta-sheet twisting (V144-Y146). Determination of the 3D structure of the Rv0679c protein could help the future study. The ladder-like patterns in BN-PAGE, resulting from the self-aggregation, differed between the N- and K-types, which migrated in a similar pattern in SDS PAGE. The propensity for aggregation was lesser in K-type than in N-type, consistent with a previous study that recombinant *Escherichia coli* of Rv0679c K-type expression was less aggregating compared to the Rv0679c N type recombinant *Escherichia coli* under isopropyl β-D-1-thiogalactopyranoside stimulation [23]. However, the process by which the altered propensity for aggregation and resulting Asn142Lys substitution-led change in antigenicity affects survival of the Beijing genotype MTB is ascertained. 8G10-H2 failed to recognize synthetic peptides as antigens (Fig. 1c), and 8G10-H2 recognized conformational epitopes of Rv0679c N-type in structure restricted western blot [14]. Our speculation was that the Asn142Lys substitution plays a role in the escape of MTB from the host immune response, as K-type-specific mAbs have not been observed in Balb/c mice. However, future experiment by using mutated MTB challenge model will be in need to verify the speculation. Importantly, we observed recognition of K-type-specific epitopes in this study using human plasma samples, and antibody recognizing conformational epitopes of Rv0679c K-type may also exist (Fig. 6) in human.

To characterize the antigenicity of Rv0679c and investigate K-type-specific antibody response in humans, we measured anti-Rv0679c antibody levels in subjects from China and the Philippines. Anti-TBGL antibody served as a control antibody to confirm and compare ATB status in C-PTB and P-PTB (Fig. 2). Anti-TBGL antibody titers have been reported to correlate with age but not gender [24]. Even though the mean age of C-PTB was higher than that of P-PTB (Table 2), no significant difference in titers of anti-TBGL antibody was observed between them. However, a larger proportion of P-HC than C-HC appeared positive for anti-TBGL IgG and IgA, suggesting a larger extent of latent TB infection (LTBI) in the Filipino population and could be due to frequent exposure to various antigens [25]. However, LTBI frequency in P-HC and C-HC was unconfirmed in this study. In a previous study, we reported high titers of TBGL antibodies in subjects at high TB exposure risk in China [26]. Although, it is difficult to determine whether P-HC are more exposed to TB than C-HC, we attempted to avoid the occurrence of high antibody titers due to LTBI by drawing the cutoff of anti-Rv0679c antibody titers within C-HC. Another reason to build cut-off based on C-HC is the high frequency of males in C-HC, C-PTB, and P-PTB, while P-PTB included a significantly high number of female (Table 2). Elevated anti-N-type IgG responses were observed in samples from the Philippines (Fig. 3a) in contrast to the elevated anti-K-type IgG responses observed in C-PTB (Fig. 3b). Since the Beijing and non-Beijing genotypes of MTB are predominant in China [12] and the Philippines, respectively [27], the anti-K-type IgG response in C-PTB and anti-N-type IgG response in P-PTB may correspond to the Beijing genotype MTB and non-Beijing genotype MTB, respectively. More than half the C-PTB samples were positive for anti-K-type IgG and a small number of those samples were positive for anti-N-type IgG, corresponding most likely to responses to Beijing genotype MTB infections and minor non-Beijing genotype MTB infections in China, respectively (Fig. 4a). The anti-N-type and anti-K-type IgG responses constitute a pair of antithetical responses to a specific antigen pertaining to non-Beijing or Beijing genotype MTB (Fig. 4a), suggesting a unique response to either Asn142 or Lys142. In a previous study with Japanese samples, the majority of samples from both non-Beijing and Beijing groups failed to react to both types of Rv0679c [15]. Unlike samples from Japan, which is a TB low burden country, the majority of samples from China responded to Rv0679c. High MTB antigen-IgG avidity in subjects from countries with high TB infection burden has also been reported in another study [28]. In this study, we did not isolate bacilli from each C-PTB sample and were, therefore, unable to demonstrate the genotype. However, all sputum samples of P-PTB were confirmed to be Manila Type (SIT 19, SpolDB4) non-Beijing strains infection by sputum spoligotyping [27]. On the other hand, the proportion of anti-K-type IgG (53.3%) and anti-N-type IgG (6.67%) was found to be less than the approximate proportion of the distribution of the Beijing (70%) and non-Beijing (30%) genotypes of MTB infection in China, indicating that nearly 20% subjects did not react to Rv0679c (Fig. 4a).

Approximately 5 of the 15 amino acids that are involved in the spatial contact of antibody and epitope strongly influence the binding [29]. Certain substitutions at any of these strong sites can change the relative binding constant by two or three orders of magnitude [30]. The Asn to Lys substitution, focused on in the present study, may be located at one of the strong sites in association with antibody recognition strength, in which antibodies that were stimulated by Lys142 of the Beijing genotype failed to recognize the Asn142 antigen of the non-Beijing genotype. However, in mice, N-type immunization did not lead to K-type recognition, while K-type immunization led to N-type recognition. Moreover, a previous studies reported that a Lys to Ala substitution of a single on Acl-11 of myelin basic protein stimulates the production of stronger autoimmune T cells [31]. An altered immune response has also been reported after substitution of three charged amino acid residues at positions 151, 153, and 154 within the C protein, conserved among Sendai virus strains, and at positions 52, 321, 351, 352, 371, 373, 375, and 377 within the nucleoprotein in Influenza virus [32, 33]. These amino acid substituted peptide antigens generate a response by T-dependent B cells, which play a role in antibody production [34, 35], while the strength of cross-reactivity between antigens decreases linearly with the number of amino acid substitutions when measured by polyclonal sera of the full immune response [36]. The cross-reaction of polyclonal antibodies may not be decreased in vitro by only a single amino acid substitution at position 142 of Rv0679c, considering that the linear epitopes of the target protein are located at position 38–48 [8], which explains the similar pattern of antibody reaction observed in response to both N-type and K-type in participants from the Philippines. However, since the Asn142Lys mutation has the ability to bring about conformational changes in Rv0679c (Fig. 1e), those infected by the Beijing genotype of MTB carry antibodies against the conformational epitope of Rv0679c-Lys142 and would show reduced response to Rv0679c-Asn142. For the first time, we reported that one amino acid substitution can result in different antibody responses in TB infection; however the importance of such substitution is still not fully known.

In spite of the higher reactivity of both N-type and K-type IgA in samples from the Philippines than C-PTB from China (Fig. 3c and d), IgA appeared to react against N/K-types equally in each group (Fig. 4b and d; *p* > 0.05 for both N- and K-types). Similar IgA reactivity patterns were confirmed by correlation analysis (Fig. 5b, *p* < 0.0001 for both C-PTB and P-PTB). However, the anti-K-type IgA and IgG responses were not correlated in C-PTB and P-PTB samples (Fig. 5d, panels L and R). Elevated specific IgA in the absence of specific IgG was also observed in a study on Rv2018 [37]. Antigens of *Plasmodium falciparum* were reported to induce the production of a specific IgG subclass in humans [38]. An epitope-specific IgG class switch was also reported in a study of malarial merozoite surface protein induction of IgG2b in mice [39]. Therefore, IgA response to K-type in the absence of an IgG response may be associated with an epitope-dependent immunoglobulin class switch event. Rv0679c-specific IgA and IgG responses may be the result of induction by conserved and dimorphic epitopes, respectively. To confirm that immunoglobulin production depends on the Asn142Lys mutation in Rv0679c, the subclass of immunoglobulins expressed needs to be determined.

Of note, we did not observe differences between P-PTB and P-HC for anti-Rv0679c IgG or IgA (Fig. 3), probably due to LTBI in the majority of P-HC [25]. Interestingly, we found an inverse correlation between the mean age of P-HC and Rv0679c antibody responses (Table 3), in contrast to a positive correlation between the age and TBGL antibody production in these participants. Cifuentes et al. have reported the invasive effect of Rv0679c in non-Beijing MTB and proposed a protective effect of Rv0679c antibodies [13], however the protective effect of Rv0679c antibodies was not evaluated. Therefore, our results may provide insight into the likely declining protective effect of Rv0679c with increasing age in P-HC from endemic regions of non-Beijing genotypes of MTB [40]. This protection against non-Beijing genotype MTB may be raised owing to BCG vaccination (Rv0679c-Asn142); the fading protective effect of BCG has been reported previously [41, 42]. The anti-Rv0679c antibodies in P-HC could target the un-mutated region of Rv0679c of both Beijing or non-Beijing genotypes of MTB, and their production may be stimulated by BCG-specific memory immune cells in response to non-Beijing MTB challenge [28]. Unlike the anti-TBGL response that appeared in ATB infected rather than BCG vaccinated individuals [27], it is indistinguishable whether the raised anti-Rv0679c IgG responses are due to BCG vaccination (P-HC) or non-Beijing TB infection (P-PTB). Since Beijing genotype MTB infection has rarely been reported in the Philippines [43], the BCG vaccine might work effectively on preventing HC from developing ATB. Moreover, mucosal IgA showed MTB blocking activity independently of Fc receptor expression, whereas IgG antibodies promoted the host cell infection, indicating higher IgA instead of IgG in the P-HC possessed an inhibitory effect against MTB [44]. Therefore, a design for a cross protective form, which can induce protective isotype antibody, based on antigenic difference Rv0679c Asn142Lys should be considered [14].

**Table 3:** Correlation between age and TB-related antibody responses

TB-related antibody test		AGE			
		C-PTB	C-HC	P-PTB	P-HC
TBGL	Anti-TBGL IgG	0.37 (0.012)^a^	0.42 (0.007)	n.s	0.66 (0.043)
	Anti-TBGL IgA	n.s	n.s	n.s	0.37 (<0.0001)
Rv0679c	Anti-N-type IgG	n.s	n.s	n.s	−0.49 (0.006)
	Anti-K-type IgG	n.s	n.s	n.s	−0.53 (0.003)
	Anti-N-type IgA	n.s	n.s	n.s	−0.39 (0.031)
	Anti-K-type IgA	n.s	n.s	n.s	−0.44 (0.016)

^a^indicates that *r* (*p*) calculated as Spearman’s rank correlation coefficients; *p* < 0.05 indicates a significant correlation

## Conclusions

In conclusion, different patterns of IgG and IgA responses against dimorphic Rv0679c were described, for the first time, in this study. A high N-type IgG reactivity in TB patients in China, where the Beijing genotype of MTB is predominant, suggests a Beijing genotype-specific antibody response. The antithetical N-type and K-type IgG responses in patients from China and equal N-type and K-type responses in patients from the Philippines suggest that the missense mutation at nucleotide 426 in *Rv0679c* results in antigenic changes. Specific IgG antibodies may be produced against the variable epitope arising from the Asn142Lys substitution, whereas IgA can be induced against the invariable epitope. Moreover, approximately 20% of samples did not respond to either N-type/K-type, indicating that the use of only a pair of mutated proteins is not adequate for Beijing/non-Beijing genotype MTB diagnosis. Therefore, more proteins specific for strains belonging to the Beijing genotype of MTB could be utilized to increase the possibility of positive samples. The major limitations of this study are indetermination of strain genotype in the Chinese ATB patients, and undefined LTBI state in the HC who are at high risk to tuberculosis. Therefore, Future studies using larger samples and in other countries need to be conducted to standardize the ELISA in terms of discrepancy in serum processing conditions, possibility of antigen exposure, and total IgG titers.

## Abbreviations

ATB: Active tuberculosis
BCG: Bacillus Calmette–Guérin
ELISA: Enzyme-linked immunosorbent assay
HC: Healthy controls
LTBI: Latent TB infection
mAb: Monoclonal antibody
MTB: Mycobacterium tuberculosis
PTB: Pulmonary tuberculosis
SHAPHC: Shanghai Public Health Clinical Center
TB: Tuberculosis
WHO: World Health Organization
